# Reprogramming of fibroblasts into cancer-associated fibroblasts via IGF2-mediated autophagy promotes metastasis of lung cancer cells

**DOI:** 10.1016/j.isci.2024.111269

**Published:** 2024-10-28

**Authors:** Limin Cao, Bingbing Li, Sijia Zheng, Qicheng Zhang, Yongmei Qian, Yinghui Ren, Huimin Wang, Min Wang, Xiang Wu, Jiayi Zhang, Ke Xu

**Affiliations:** 1 Tianjin Key Laboratory of Lung Cancer Metastasis and Tumor Microenvironment, Tianjin Lung Cancer Institute, Tianjin Medical University General Hospital, Tianjin 300052, China; 2 Department of Anesthesiology, Tianjin First Central Hospital, Tianjin 300192, China; 3 Department of Clinical Laboratory, Tianjin First Central Hospital, Tianjin 300192, China; 4 Core Facility Center, Tianjin Medical University General Hospital, Tianjin 300052, China

**Keywords:** Cell biology, Cancer

## Abstract

Cancer-associated fibroblasts (CAFs) are major component of stromal cells. Growing evidence suggests that CAFs promote tumor growth and metastasis; however, the reprogramming of normal fibroblasts (NFs) into CAFs by tumor cells still remains largely unknown. In this study, we found that non-small cell lung cancer (NSCLC) cells activated NFs into CAFs via autophagy induction. Insulin-like growth factor 2 (IGF2) secreted by NSCLC cells mediated NSCLC cells’ effect on autophagy induction and CAFs activation. Importantly, the activated CAFs promoted NSCLC cells growth, migration, and invasion. Further study showed that the activated CAFs facilitated NSCLC cells invasion via promoting epithelial-mesenchymal transition (EMT) process, upregulating metastasis-related genes, releasing CXCL12, and activating its downstream AKT serine/threonine kinase 1 (AKT)/ nuclear factor κB (NF-κB) signaling pathway. These findings revealed that IGF2-mediated autophagy plays a critical role in CAFs activation and suggested the IGF2-autophagy cascade in fibroblasts could be a potential target for lung cancer therapy.

## Highlights

• NSCLC cells activate NFs into CAFs via autophagy induction
• IGF2 secreted by NSCLC cells mediates NSCLC cells’ effect on CAFs activation
• iCAFs promote NSCLC cells growth, migration, and invasion
• iCAFs facilitated NSCLC cells invasion via promoting EMT and releasing CXCL12

## Graphical abstract

**Figure:**
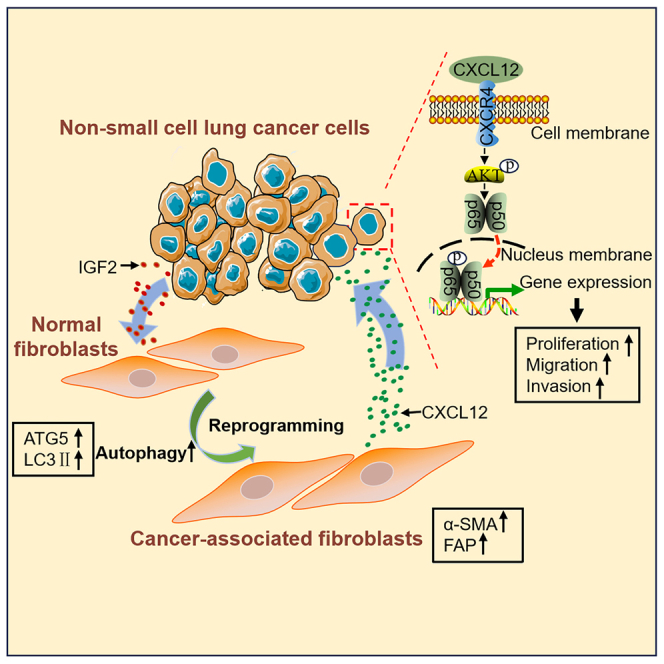

## Introduction

Lung cancer is the most lethal cancer in the world, with an estimated 1.8 million deaths each year (18% of total cancer deaths).^1^ The major pathological subtype of lung cancer is NSCLC, which accounts for about 85% of total lung cancer. Lung cancer has high metastatic capacity; nearly 90% of lung cancer patients die of metastasis.^2^ Despite the significant achievement of early diagnosis and treatment, the outcome of lung cancer treatment is not optimistic; the 5-year survival rate is under 20%.^3^ Thus, an in-depth understanding of the mechanism of lung cancer metastasis is urgently needed for the development of novel therapeutic strategy.

Accumulating evidence shows that the tumor microenvironment (TME) plays a pivotal role in the initiation and progression of tumor.^4^ Cancer-associated fibroblasts (CAFs) are major stromal cells in the TME. Although there are no specific markers for CAFs, CAFs usually display high level of several proteins such as α-smooth muscle actin (α-SMA), fibroblast-specific protein 1 (FSP1), and fibroblast-activating protein (FAP)^5^; among them α-SMA is the most commonly used marker. CAFs facilitate tumor growth and metastasis by remodeling the extracellular matrix, interacting with adjacent tumor cells, and releasing a variety of molecules such as growth factors and cytokines to promote cell proliferation, migration, and invasion.^6^ CAFs induce growth and radioresistance of breast cancer cells via interleukin-6 (IL-6) secretion.^7^ Periostin derived from CAFs promotes progression of esophageal squamous cell carcinoma through ADAM17 activation.^8^ Exosomal miR-146b-5p derived from CAFs facilitates oral squamous cell carcinoma progression by downregulating HIPK3.^9^ Our previous studies also demonstrate that CAFs enhance lung cancer metastasis via secreting HMGB1, VEGFA, KRT8, and IL-6.^10^^,^^11^^,^^12^^,^^13^

The communications between tumor cells and CAFs play an essential role in tumor development. There are intensive studies focused on this; however, how tumor cells activate normal fibroblast (NF) still remains largely unknown. The understanding of the underlying mechanism of CAFs activation will provide new insight into cancer therapy by targeting the TME.

Autophagy is a primary intracellular recycle mechanism. In response to stresses such as starvation and drug treatment, autophagy stimulates the degradation and recycle of cellular cargos to provide the resource for proteins and lipids synthesis in order to maintain cellular homeostasis. Autophagy is involved in cell death, differentiation, energy metabolism, and organ homeostasis. Numerous studies show that autophagy plays a key role in tumor development; in particular, it plays dual roles in metastasis. Autophagy facilitates metastasis by activating pathways for cell migration and invasion, stimulating epithelial-mesenchymal transition (EMT) process, and adapting cells to hypoxia. On the contrary, autophagy suppresses metastasis by stimulating immune response, which leads to the elimination of cancer cells.^14^ Increasing evidence revealed that autophagy also influences CAFs functions. Autophagy regulates the secretory capability of CAFs, the metabolism of CAFs, and the signaling pathways in CAFs.^15^

Insulin-like growth factor 2 (IGF2) is a secretory protein of 67 amino acids, which belongs to the insulin growth factor (IGF) system. In the bloodstream, the plasma IGF2 is stabilized by the IGF binding proteins (IGFBPs). IGF2 mainly binds to the type I IGF receptor (IGF1R), leading to the activation of downstream signaling pathways such as mitogen-activated protein (MAP) kinase pathway and PI3K kinase pathway. IGF2 regulates cell proliferation, survival, differentiation, and metabolism. Numerous studies have shown that IGF2 is involved in tumor development and metastasis.^16^ Despite the intensive studies on the role of IGF2 in cancer, whether IGF2 is responsible for the activation of CAFs is not elucidated.

In the present study, we found that lung cancer cells activated NFs to CAFs via autophagy induction, and IGF2-secreted by lung cancer cells mediated the autophagy induction of NFs. On the other hand, the activated CAFs facilitated the metastasis potential of lung cancer cells through CXCL12 release and downstream AKT/nuclear factor κB (NF-κB) pathway activation. Our study revealed the critical role of IGF2-mediated autophagy in the activation of CAFs, more importantly, the interplay between lung cancer cells and stromal fibroblasts in the TME, and identified IGF2 as a candidate target for lung cancer treatment.

## Results

### Lung cancer cells induce reprogramming of NFs into CAFs

**Figure 1:**
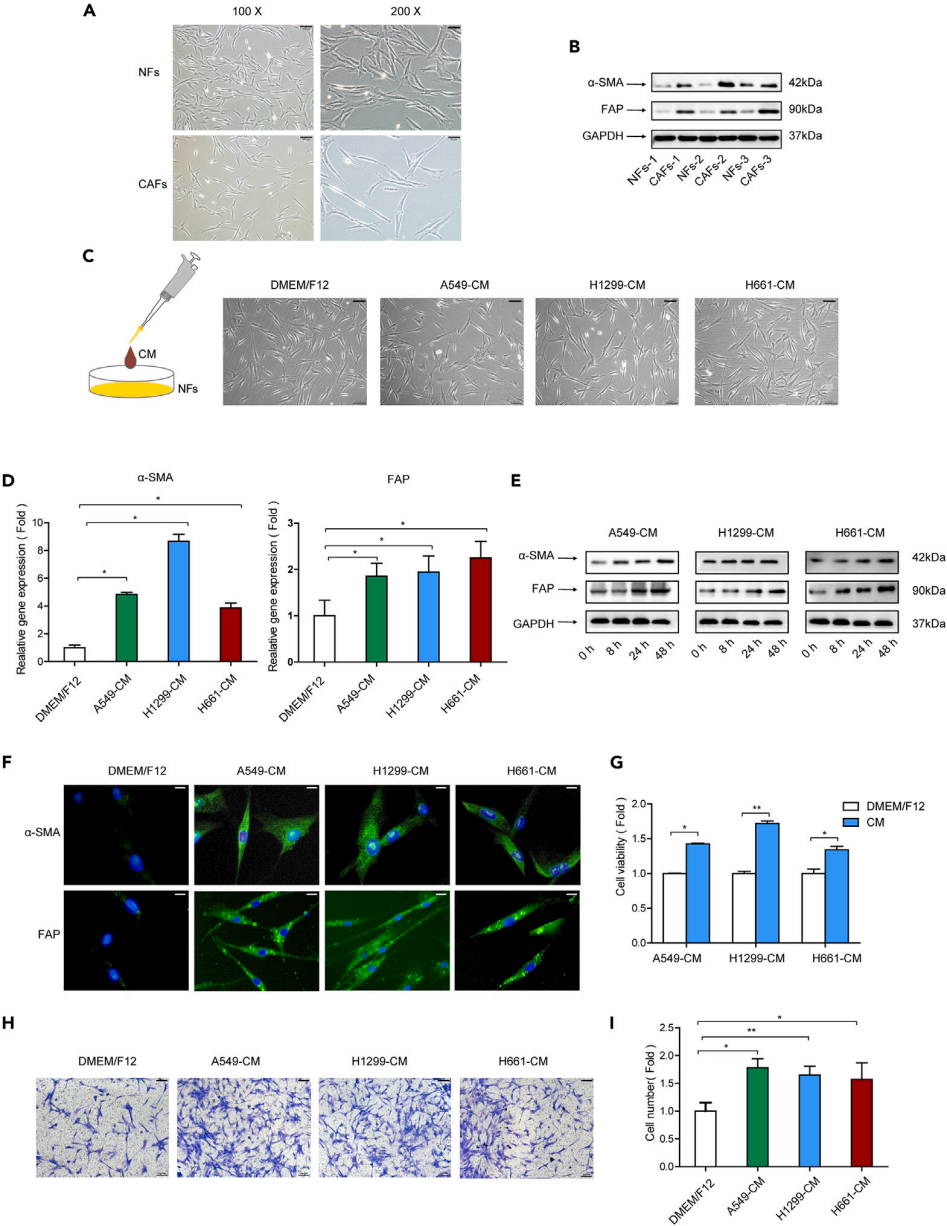
Lung cancer cells induced reprogramming of NFs into CAFs (A) Cell morphology was observed under a microscope (10X, scale bar,= 100 μm; 20X, scale bar, 50 μm). (B) α-SMA and FAP expression were detected by western blotting. (C) NFs were treated with CM. Cell morphology was observed under a microscope (scale bar, 100 μm). (D) NFs were treated with CM. α-SMA and FAP expressions were detected by qPCR. (E) NFs were treated with CM. α-SMA and FAP expressions were detected by western blotting. (F) NFs were treated with CM. α-SMA and FAP expressions were detected by immunofluorescence staining (scale bar, 20 μm). (G) NFs were treated with CM. Cell proliferation was examined by CCK-8 kit after 48 h. (H and I) NFs were treated with CM. NFs migration was detected by trans-well assay after 24 h (scale bar, 100 μm). Data represents the mean ± SD from three independent experiments. *∗p < 0.05, ∗∗p < 0.01, ∗∗∗p < 0.001*.

In order to investigate the activation of NFs to CAFs, we first compared the characters of NFs and CAFs. Three pairs of CAFs and NFs were isolated from lung cancer tissues and matched adjacent non-tumor tissues. The morphology observation indicated that both NFs and CAFs displayed a thin and spindle-like appearance, and there was no significant difference between these two types of cells (Figure 1A). We then examined the common markers for CAFs. As shown in Figure 1B, CAFs expressed higher levels of α-SMA and FAP than NFs.

To assess the effect of lung cancer cells on CAFs activation, the conditioned medium (CM) was collected from three lung cancer cell lines (A549, H1299, H661) and added to NFs culture; then CAFs markers were examined. Although the cell morphology did not change after CM treatment (Figure 1C), both α-SMA and FAP were upregulated at both the mRNA level and protein level (Figures 1D and 1E); this was confirmed by immunofluorescence staining (Figure 1F). Because activated CAFs are reported to proliferate faster and more invasive than NFs,^17^^,^^18^ we further measured these cell activities. After CM treatment, the growth and migration of NFs were boosted dramatically (Figures 1G–1I). Taken together, our results demonstrated that lung cancer cells reprogrammed NFs into CAFs, and we named these activated NFs as induced CAFs (iCAFs).

### Lung cancer cells induce CAFs activation via IGF2 secretion

**Figure 2:**
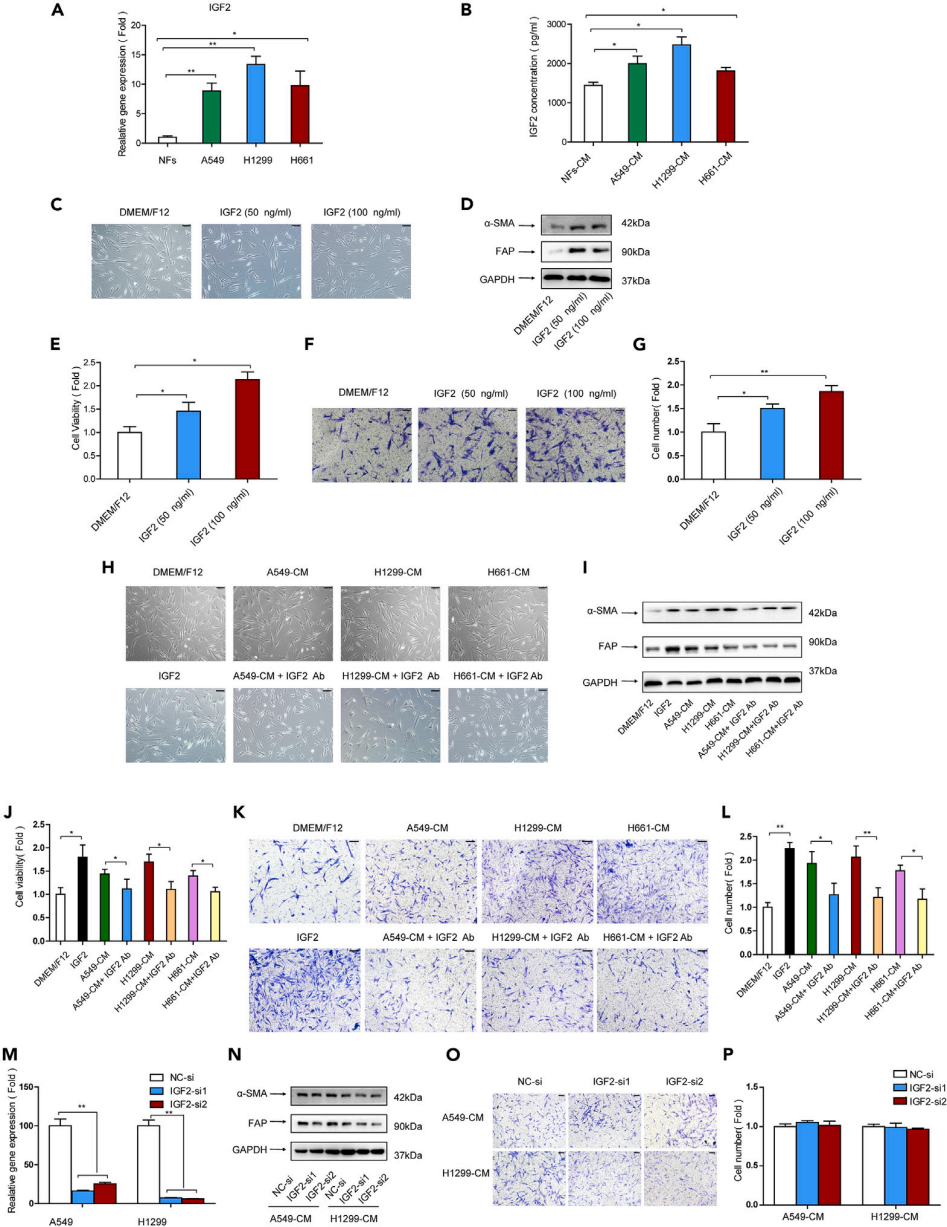
Lung cancer cells induced CAFs activation via IGF2 secretion (A) mRNA levels of IGF2 in cells were detected by qPCR. (B) IGF2 concentrations in cell culture medium were detected by ELISA. (C) NFs were treated with IGF2 (50 and 100 ng/mL) for 24 h. Cell morphology was observed under a microscope. (D) NFs were treated with IGF2. α-SMA and FAP expressions were detected by western blotting. (E) NFs were treated with IGF2. Cell proliferation was examined by CCK-8 kit after 48 h. (F and G) NFs were treated with IGF2. NFs migration was detected by trans-well assay after 24 h. (H) NFs were treated with CM or CM + IGF2 neutralizing antibody. Cell morphology was observed under a microscope. (I) NFs were treated with CM or CM + IGF2 neutralizing antibody. α-SMA and FAP expressions were detected by western blotting. (J) NFs were treated with CM or CM + IGF2 neutralizing antibody, and IGF2 (100 ng/mL). Cell proliferation was examined by CCK-8 kit after 48 h. (K and L) NFs were treated with CM or CM + IGF2 neutralizing antibody. NFs migration was detected by trans-well assay after 24 h. (M) The effect of IGF2 knockdown by RNAi. (N) IGF2 expression was knocked down in lung cancer cells, and then the CM was collected and used to culture NFs. α-SMA and FAP expressions were detected by western blotting. (O and P) IGF2 expression was knocked down in lung cancer cells by RNAi, and then the CM was collected and used to culture NFs. NFs migration was detected by trans-well assay after 24 h. Ab, neutralizing antibody. Scale bar, 100 μm. Data represents the mean ± SD from three independent experiments. *∗p < 0.05, ∗∗p < 0.01, ∗∗∗p < 0.001*.

Evidence has demonstrated that tumor cells can release growth factors or cytokines to transform NFs to CAFs.^19^ Since IGF2 is reported to be responsible for tumor metastasis,^16^ and CAFs play key role in metastasis, this drove us to investigate whether IGF2 is involved in CAFs activation. First, we compared the IGF2 level in lung cancer cells and fibroblasts. IGF2 mRNA levels were detected by qPCR, and secreted IGF2 were measured by ELISA. Figures 2A and 2B showed that the IGF2 levels in lung cancer cells were significantly higher than those in NFs. Next, we tested whether IGF2 is the main driver of CAFs activation. We added recombinant human IGF2 to NFs cultures at the concentrations of 50 and 100 ng/mL and assessed the activation of NFs.^20^ The morphology of NFs was not changed by IGF2 (Figure 2C); however, the expressions of CAFs markers α-SMA and FAP were increased dramatically, and cell viability and migration ability were enhanced in a dose-dependent manner (Figures 2D–2G). These data indicated that IGF2 may activate NFs.

Since we found that lung cancer cells possessed higher level of IGF2 than NFs (Figure 2B), this implied the possibility that lung cancer cells reprogram NFs via IGF2 secretion. In order to demonstrate this hypothesis, neutralizing antibody against IGF2 was added to lung cancer cell-CM and to culture NFs. There were no obvious changes in NFs morphology (Figure 2H); interestingly, we found that the stimulating effects of CM on CAFs markers expression, cell viability, and cell migration were attenuated significantly by IGF2 blocking (Figures 2I–2L). To further confirm this finding, IGF2 expression was knocked down in lung cancer cells by RNAi, and then the CM was collected and used to culture NFs. As expected, IGF2 knockdown in lung cancer cells mitigated the promoting effect of lung cancer cell-CM on CAFs markers expression and cell migration (Figures 2M–2O). Collectively, our data provided the first evidence that lung cancer cells activated NFs to iCAFs via IGF2 secretion.

### IGF2-induced autophagy mediates the effect of lung cancer cells on CAFs activation

**Figure 3:**
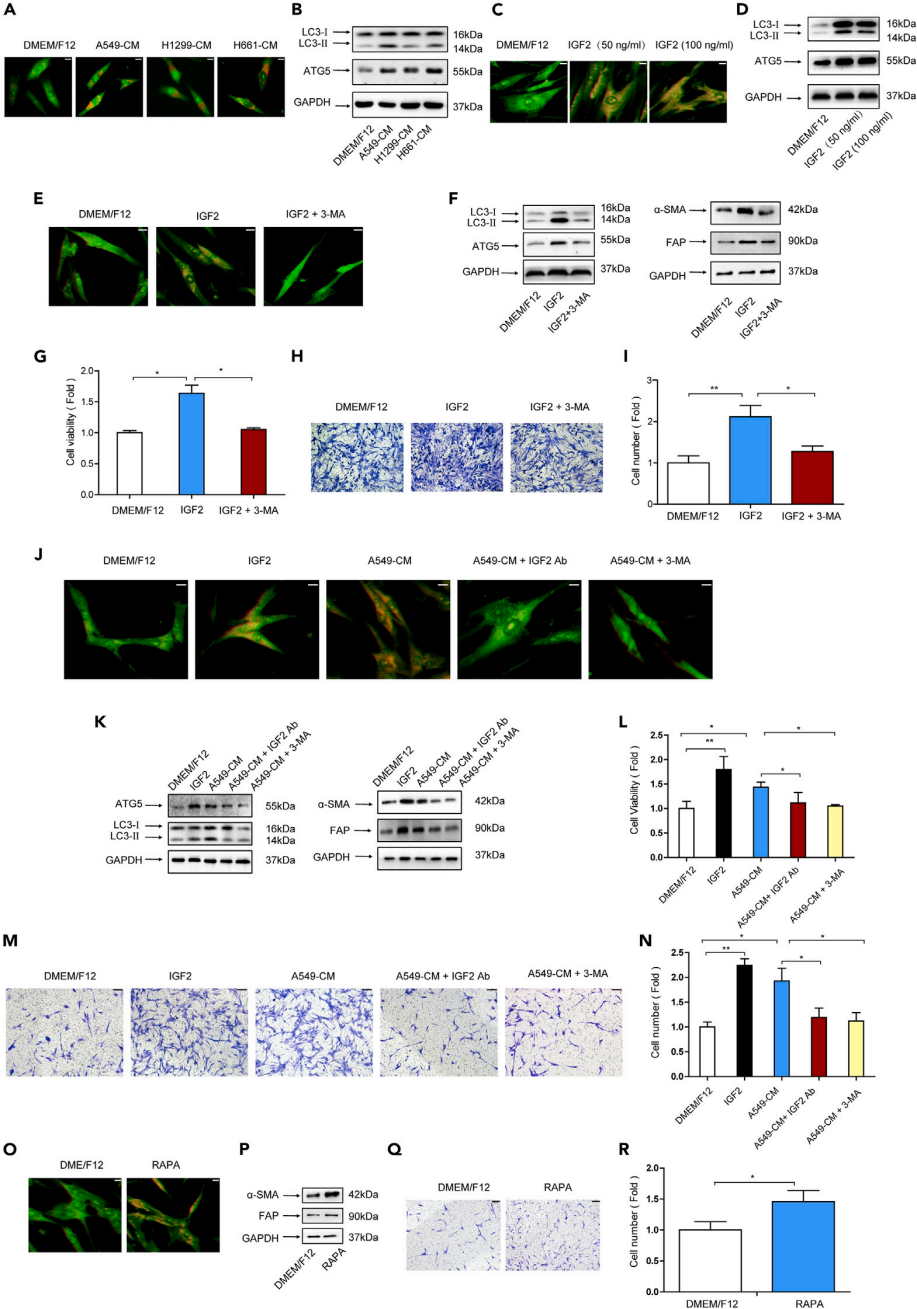
IGF2-induced autophagy mediated the effect of lung cancer cells on CAFs activation (A) NFs were treated with CM and then were stained with 0.5 μg/mL AO for 15 min. The acidic vesicular organelles (AVOs) formation was observed under a fluorescence microscope (scale bar, 20 μm). (B) NFs were treated with CM. The expression of LC-3II and ATG5 were detected by western blotting. (C) NFs were treated with IGF2 (50 or 100 mg/mL) for 24 h. The AVOs formation was examined by AO staining (scale bar, 20 μm). (D) NFs were treated with IGF2. The expression of LC-3II and ATG5 was detected by western blotting. (E) NFs were treated with IGF2 or IGF2 + 3-MA (5 mM). The AVOs formation was examined by AO staining (scale bar, 20 μm). (F) NFs were treated with IGF2 or IGF2 + 3-MA. Protein expressions were detected by western blotting. (G) NFs were treated with IGF2 or IGF2 + 3-MA. Cell proliferation was examined by CCK-8 kit after 48 h. (H and I) NFs were treated with IGF2 or IGF2+3-MA. NFs migration was detected by trans-well assay after 24 h (scale bar, 100 μm). (J) NFs were treated with IGF2, CM, CM + IGF2 Ab, and CM + 3-MA. The AVOs formation was examined by AO staining (scale bar, 20 μm). (K) NFs were treated with CM, IGF2, and CM + IGF2 Ab. Protein expressions were detected by western blotting. (L) NFs were treated with IGF2, CM, CM + IGF2 Ab, and CM + 3-MA. Cell proliferation was examined by CCK-8 kit after 48 h. (M and N) NFs were treated with IGF2, CM, CM + IGF2 Ab, and CM + 3-MA. NFs migration was detected by trans-well assay after 24 h (scale bar, 100 μm). (O and P) NFs were treated with RAPA (100 nM); α-SMA and FAP expressions were detected by western blotting (scale bar, 20 μm). (Q and R) NFs were treated with RAPA; NFs migration was detected by trans-well assay after 24 h. Ab, neutralizing antibody (scale bar, 100 μm). Data represents the mean ± SD from three independent experiments. *∗p < 0.05, ∗∗p < 0.01, ∗∗∗p < 0.001*.

Our previous study showed that CAFs possess a higher level of autophagy than NFs, and autophagic secretion of HMGB1 from CAFs promotes metastasis of lung cancer cells.^10^ Thus, we explored the role of autophagy in CAFs activation. CM collected from 3 lung cancer cell lines was used to culture NFs, and the autophagy level of NFs was evaluated. The formation of AVOs is a hallmark of autophagy, and the results of acridine orange (AO) staining of AVOs revealed that CM elevated autophagy level of NFs (Figure 3A). We next examined the expression of autophagy-related genes LC3-II and ATG5. Both genes were upregulated by CM (Figure 3B). These data suggested that lung cancer cells induced autophagy of NFs.

Given that lung cancer cells induced CAFs activation via IGF2 secretion, we wondered whether IGF2 could induce autophagy of NFs. Figures 3C and 3D showed that IGF2 increased AVOs formation and LC3-II and ATG5 expression. Notably, when autophagy inhibitor 3-MA was applied,^10^ the autophagy induction and CAFs activation in terms of the CAFs markers and enhancement of cell proliferation and migration by IGF2 were abolished (Figures 3E–3I), suggesting that IGF2 could induce autophagy of NFs and it was critical for IGF2-mediated CAFs activation. To further investigate whether IGF2 is responsible for the autophagy induction of NFs by lung cancer cells, IGF2 neutralizing antibody was added to CM. As shown in Figures 3J–3N, IGF2 blockage attenuated autophagy induction and also the stimulation of cell proliferation and migration of NFs by lung cancer cells.

To verify the role of autophagy in CAFs activation by lung cancer cells, autophagy inhibitor 3-MA was supplied in CM and cultured NFs. We found that autophagy inhibition attenuated CAFs activation by lung cancer cells (Figures 3J–3N). The role of autophagy was further confirmed by elevating autophagy level through treating NFs with autophagy agonist rapamycin (RAPA).^10^ Figures 3O–3R indicated that autophagy stimulation increased the level of CAFs markers and cell migration. Altogether, our results indicated that IGF2-induced autophagy mediated the effect of lung cancer cells on CAFs activation.

### iCAFs promote migration and invasion of lung cancer cells

**Figure 4:**
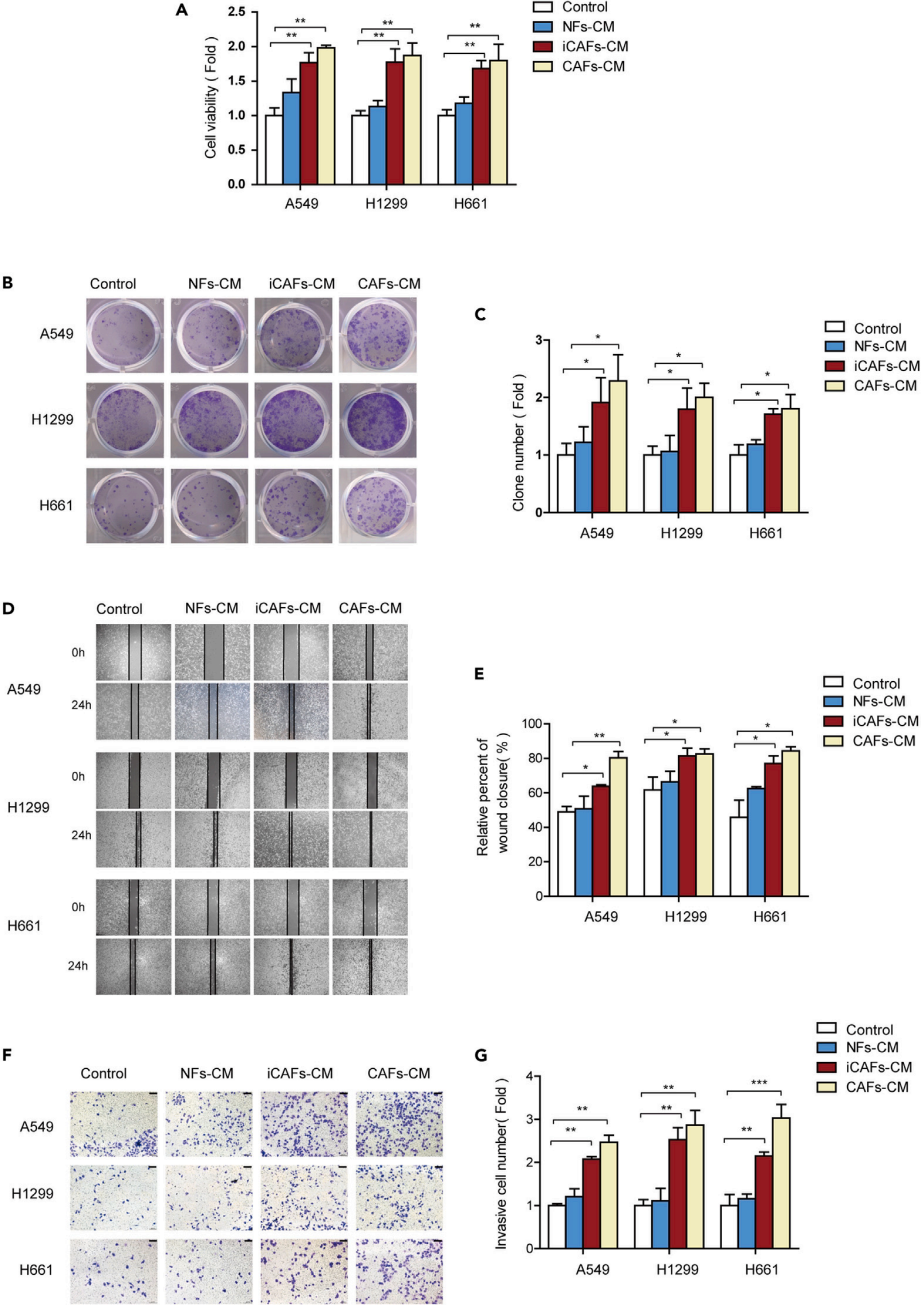
iCAFs promoted migration and invasion of lung cancer cells (A) Lung cancer cells were treated with CM. Cell proliferation was examined by CCK-8 kit after 48 h. (B and C) Cell proliferation was detected by colony formation. (D and E) Cell migration was detected by wound healing assay after 24 h. (F and G) Cell invasion was detected by trans-well assay after 24 h (scale bar, 100 μm). Data represents the mean ± SD from three independent experiments. *∗p < 0.05, ∗∗p < 0.01, ∗∗∗p < 0.001*.

Our previous studies demonstrated that CAFs facilitate the metastasis of lung cancer cells^10^^,^^11^^,^^13^^,^^21^; therefore, we next assessed whether iCAFs exert similar function as CAFs. The CM derived from iCAFs, NFs, and CAFs were collected to culture lung cancer cells. The effects of NFs-CM, iCAFs-CM, and CAFs-CM on cell proliferation were compared. Both cell viability assay and colony formation assay showed that both iCAFs and CAFs stimulated cell proliferation significantly; however, NFs did not enhance cell proliferation (Figures 4A–4C). To validate the effect of iCAFs on migration and invasion ability of lung cancer cells, wound healing assay and transwell assay were performed. In line with the results of cell proliferation, the CMs derived from fibroblasts enhanced migration and invasion of lung cancer cells, and iCAFs and CAFs were more effective than NFs (Figures 4D–4G). These data revealed that iCAFs exhibited similar function as CAFs, promoting growth, migration, and invasion of lung cancer cells.

### iCAFs enhance lung cancer cell migration and invasion via CXCL12-mediated AKT/NF-κB pathway

**Figure 5:**
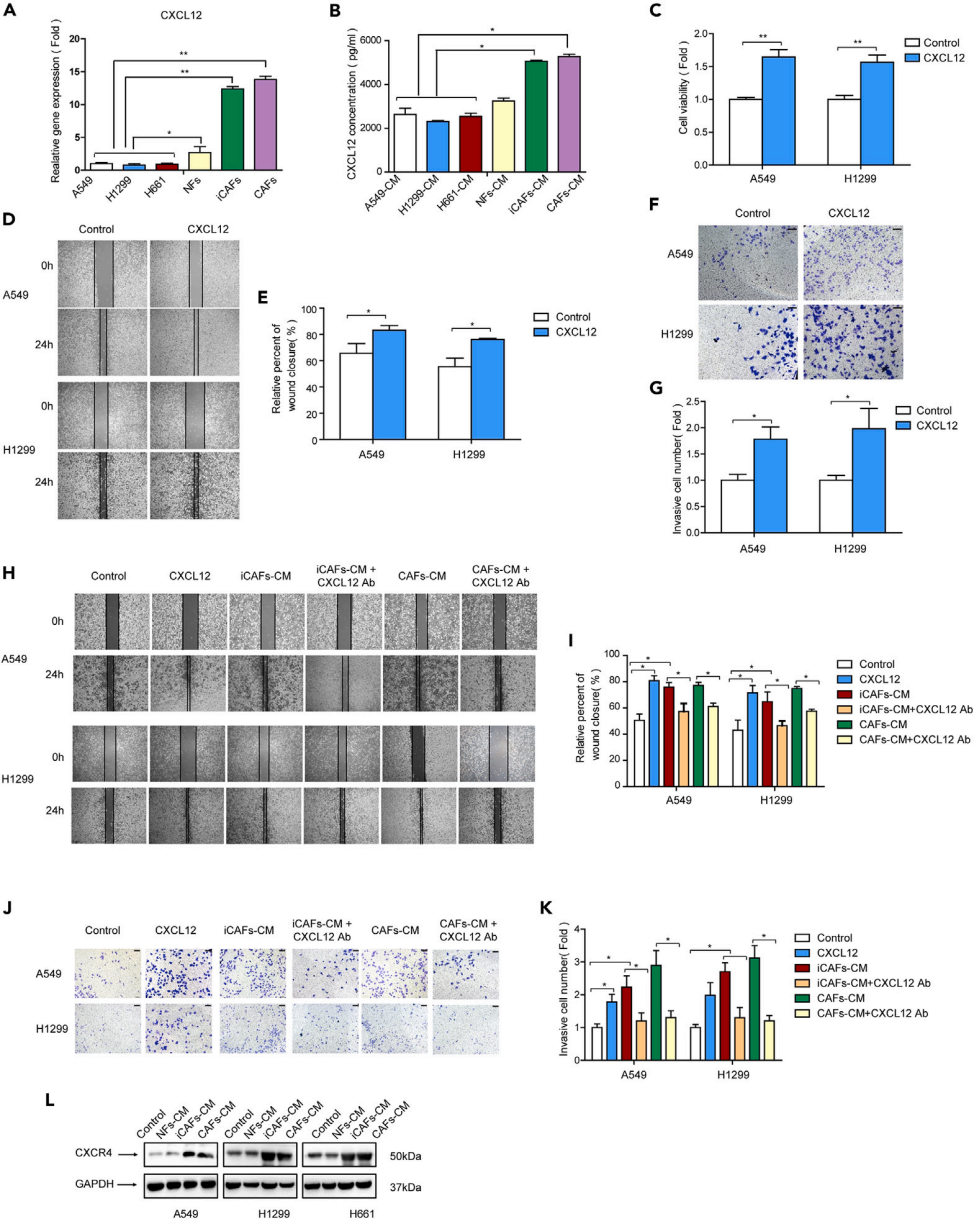
iCAFs enhanced lung cancer cell migration and invasion via CXCL12 (A) mRNA levels of CXCL12 in fibroblasts were detected by qPCR. (B) The secreted CXCL12 by cells were examined by ELISA assay. (C) Lung cancer cells were treated with CXCL12. Cell proliferation was examined by CCK-8 kit after 48 h. (D and E) Lung cancer cells were treated with CXCL12. Cell migration was detected by wound healing assay after 24 h. (F and G) Lung cancer cells were treated with CXCL12. Cell invasion was detected by trans-well assay after 24 h (scale bar, 100 μm). (H and I) Lung cancer cells were treated with CXCL12, CM, or CM + CXCX12 neutralizing antibody. Cell migration was detected by wound healing assay after 24 h. (J and K) Lung cancer cells were treated with CXCL12, CM, or CM + CXCX12 neutralizing antibody. Cell invasion was detected by trans-well assay after 24 h (scale bar, 100 μm). (L) Lung cancer cells were treated with CM. The expressions of CXCR4 were detected by western blotting. Ab, neutralizing antibody. Data represents the mean ± SD from three independent experiments. *∗p < 0.05, ∗∗p < 0.01, ∗∗∗p < 0.001*.

Next the mechanism by which iCAFs enhanced lung cancer cell migration and invasion was investigated. CAFs interact with tumor cells via secretion of various growth factors or cytokines, and several studies have shown that CAFs secrete CXCL12 to facilitate tumor progression^22^^,^^23^; this drove us to evaluate the role of CXCL12 in iCAFs’ effect on lung cancer cells. We first detected CXCL12 released from fibroblasts and lung cancer cells. The qPCR results showed that iCAFs and CAFs expressed higher level of CXCL12 than NFs, and ELISA results indicated that both iCAFs and CAFs secreted higher amount of CXCL12 than lung cancer cells (Figures 5A and 5B). Next, we studied the role of CXCL12 in cell activities by adding recombinant CXCL12 to culture medium and found that CXCL12 boosted the proliferation, migration, and invasion of lung cancer cells (Figures 5C–5G). To further validate whether CXCL12 mediated the effect of iCAFs on lung cancer cell migration and invasion, CXCL12 neutralizing antibody was applied to iCAF-CM and cultured lung cancer cells. As shown in Figures 5H–5K, recombinant CXCL12 significantly enhanced cell migration and invasion, which was similar to iCAFs and CAFs, whereas CXCL12 neutralizing antibody dramatically attenuated the stimulating effect of iCAFs. Furthermore, the expression level of CXCL12 receptor CXCR4 in lung cancer cell was detected, and we found that iCAFs elevated CXCR4 level (Figure 5L). Taken together, these results demonstrated that iCAFs promoted lung cancer cell migration and invasion via CXCL12 secretion.

**Figure 6:**
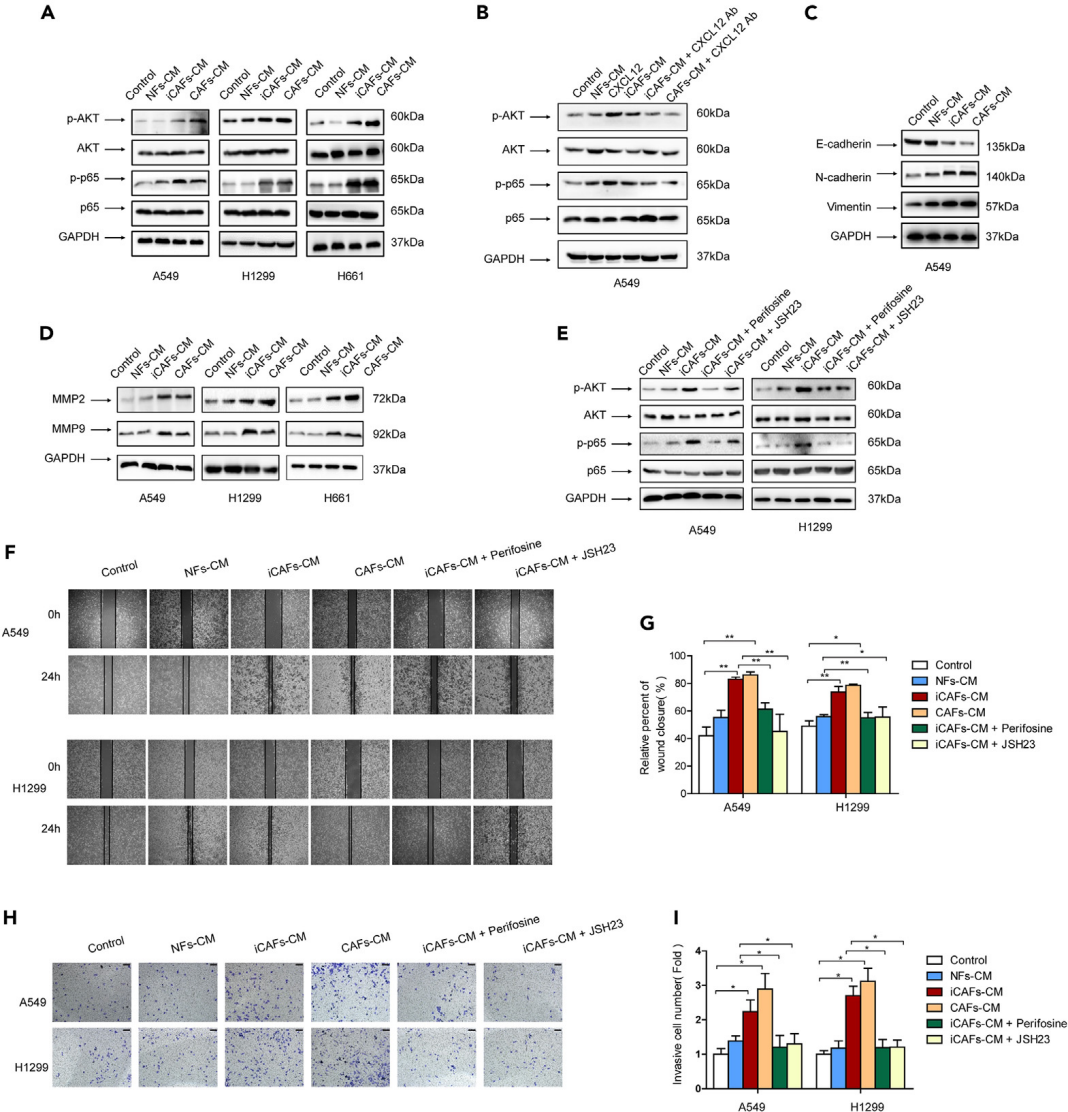
CXCL12 mediated the activation of AKT/NF-κB pathway (A) Lung cancer cells were treated with CM. Gene expressions were detected by western blotting. (B) Lung cancer cells were treated with CM, CXCL12, or CM + CXCX12 neutralizing antibody. Gene expressions were detected by western blotting. (C and D) Lung cancer cells were treated with CM. Gene expressions were detected by western blotting. (E) Lung cancer cells were pretreated with AKT inhibitor perifosine (10 μM) or NF-κB inhibitor JSH23 (10 μM) for 1 h and then treated with CM. Gene expressions were detected by western blotting. (F and G) Lung cancer cells were pretreated with AKT inhibitor perifosine (10 μM) or NF-κB inhibitor JSH23 (10 μM) for 1 h and then treated with CM. Cell migration was detected by wound healing assay after 24 h. (H and I) Lung cancer cells were pretreated with AKT inhibitor perifosine (10 μM) or NF-κB inhibitor JSH23 (10 μM) for 1 h and then treated with CM. Cell invasion was detected by trans-well assay after 24 h (scale bar, 100 μm). Ab, neutralizing antibody. Data represent the mean ± SD from three independent experiments. *∗p < 0.05, ∗∗p < 0.01, ∗∗∗p < 0.001*.

AKT/NF-κB signaling pathway is reported to be responsible for tumor metastasis.^24^^,^^25^ To explore the downstream signaling pathway of CXCL12, we examined the effect of iCAFs on AKT/NF-κB pathway. NFs-CM, iCAFs-CM, and CAFs-CM were used to culture lung cancer cells. We found that iCAFs elevated the level of p-AKT and p-p65; meanwhile, the total AKT and total p65 levels were not changed, and iCAFs had similar effect as CAFs (Figure 6A). To validate the role of CXCL12 in AKT/NF-κB pathway activation by iCAFs, recombinant CXCL12 and CXCL12 neutralizing antibody was supplied in CM and cultured lung cancer cells. Notably, CXCL12 stimulated the AKT/NF-κB pathway, whereas CXCL12 neutralizing attenuated the stimulation (Figure 6B), indicating CXCL12 mediated AKT/NF-κB pathway activation by iCAFs, EMT is a critical step for tumor metastasis. Next the EMT status was examined, and we found that iCAFs downregulated epithelial marker E-cadherin and meanwhile upregulated mesenchymal markers N-cadherin and vimentin (Figure 6C), suggesting that iCAFs facilitated EMT process. Since tumor metastasis is regulated by metastasis-related genes, we also tested the effect of iCAFs on metastasis-related genes. As shown in Figure 6D, MMP2 and MMP9 were upregulated.

Furthermore, to determine whether AKT/NF-κB signaling pathway is responsible for iCAFs’ effect on cell migration and invasion, lung cancer cells were pretreated with either AKT inhibitor perifosine or NF-κB inhibitor JSH23 and then cultured in iCAFs-CM. As shown in Figure 6E, the activation of AKT/NF-κB signaling by iCAFs was attenuated by inhibitors. Moreover, the enhancement of cell migration (Figures 6F and 6G) and invasion (Figures 6H and 6I) by iCAFs was significantly abrogated. Collectively, our data demonstrated that iCAFs enhanced lung cancer cell migration and invasion via CXCL12-mediated AKT/NF-κB pathway.

### iCAFs facilitate lung tumor growth *in vivo*

**Figure 7:**
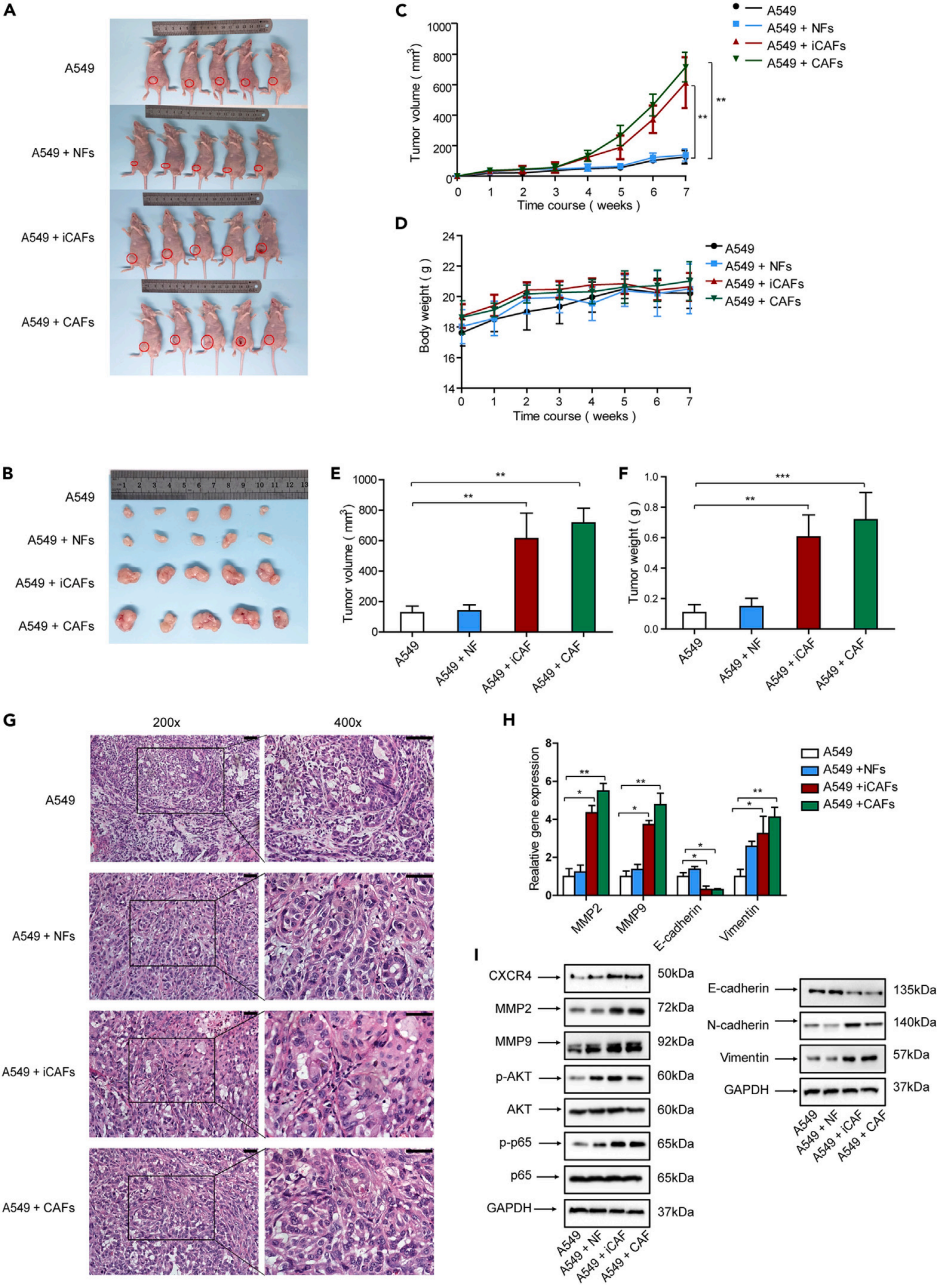
iCAFs facilitated lung tumor growth *in vivo* (A and B) Lung cancer cells were mixed with fibroblasts and injected subcutaneously into the flank of mice. Mice were grown for 7 weeks (A and B) Tumors growth for 7 weeks. (C) Tumor volumes were measured for 7 successive weeks. (D) Mice body weights were measured for successive 7 weeks. (E) Tumors were excised after 7 weeks, and tumor volumes were measured. (F) Tumors were excised after 7 weeks, and tumor weights were measured. (G) H&E staining of tumor tissues (scale bar, 50 μm). (H) Gene expressions were detected by qPCR. (I) Gene expressions were detected by western blotting. Data represent the mean ± SD from three independent experiments. *∗p < 0.05, ∗∗p < 0.01, ∗∗∗p < 0.001*.

Based on our findings that iCAFs promoted lung cancer cell growth, migration, and invasion *in vitro*, we further evaluated the role of iCAFs in tumor growth *in vivo*. The xenograft mouse models were generated by subcutaneously injecting cells into the flank of nude mice. The mice were distributed into 4 groups consisting of A549 cells alone, A549 cells + NFs, A549 cells + iCAFs, and A549 cells + CAFs. Tumor growth had been observed for 7 weeks successively. Similar to our *in vitro* results, both iCAFs and CAFs dramatically accelerated tumor growth, demonstrated by tumor volume and tumor weight. Notably, the average tumor sizes of A549 cells + iCAFs group and A549 cells + CAFs group were 4.87-fold and 5.68-fold of A549 cells alone group, respectively, indicating that the growth-promoting effect of CAFs was slightly stronger than that of iCAFs. (Figures 7A–7G).

To further reveal the underlying mechanism, we examined the CXCL12 receptor, EMT status, metastasis-related genes, and AKT/NF-κB pathway in mice tumor tissues. In line with the *in vitro* study, both qPCR and western blotting results indicated that iCAFs elevated CXCR4 level, promoted EMT process, upregulated metastasis-related genes MMP2 and MMP9, and activated AKT/NF-κB pathway (Figures 7H and 7I).

## Discussion

**Figure 8:**
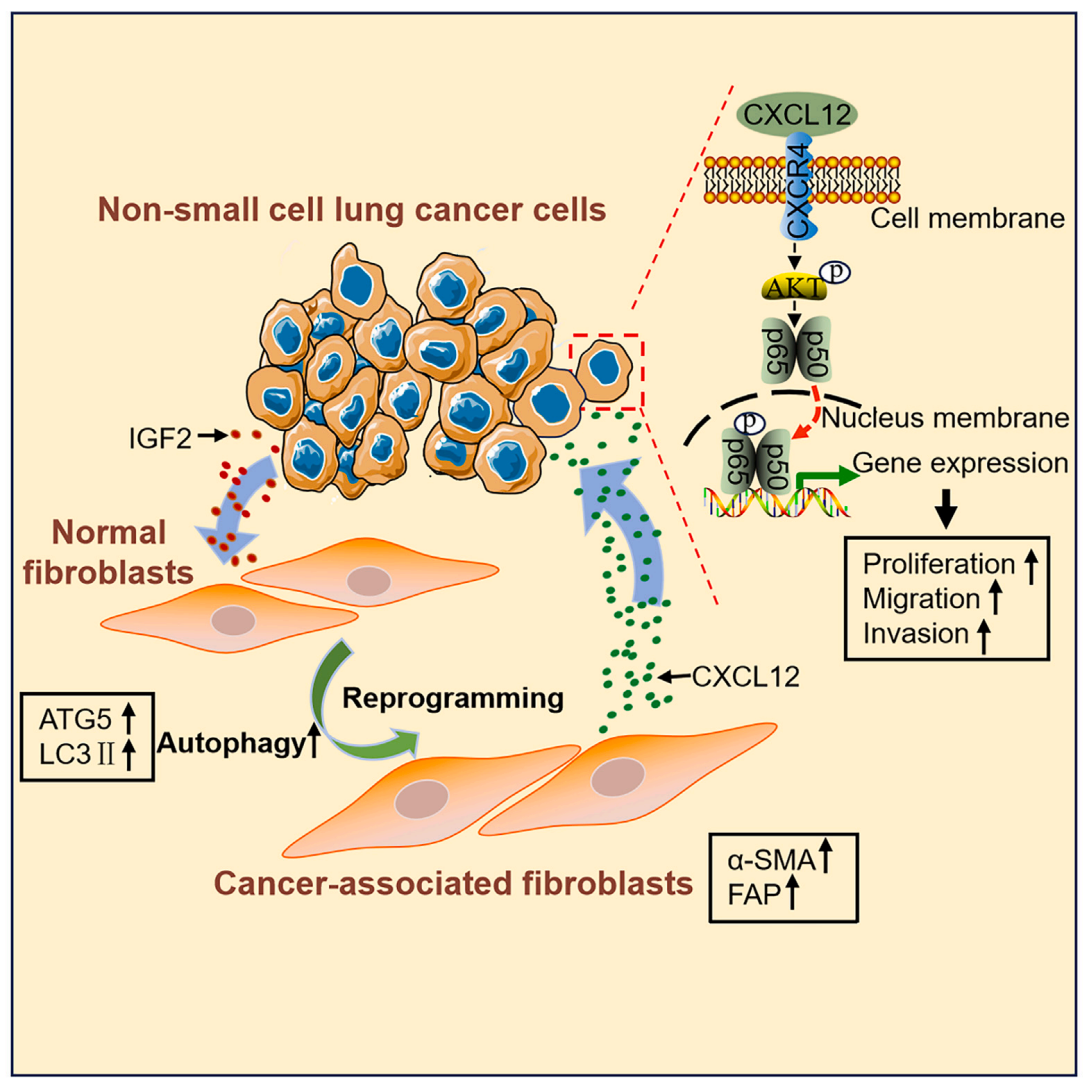
Scheme illustrating the reprogramming of NFs by lung cancer cells and the promoting effect of iCAFs on lung cancer cell metastasis

Accumulating evidence has shown that tumor development depends not only on tumor cells but also on the TME, in particular, the communication between tumor cells and stroma. CAFs are major component of stromal cells; CAFs secret growth factors and exosomes to promote malignant phenotype of tumor cells. On the other hand, tumor cells also reprogram fibroblasts into CAFs, to facilitate tumor cell growth, drug resistance, and metastasis. In this study, we demonstrated that lung cancer cells activated CAFs via IGF2-mediated autophagy induction; in return, activated CAFs stimulated lung cancer cell migration and invasion via CXCL12-mediated AKT/NF-κB pathway (Figure 8).

Recent studies reveal the role of cancer cells in CAFs activation. Among them most studies were focused on the role of cancer cell-derived extracellular vesicles (EVs). Circular RNA EHD2 (CircEHD2) from renal cell carcinoma cell-derived EVs activates CAFs to accelerate the growth of renal cell carcinoma cells via the circEHD2/YWHAH/YAP/SOX9 signaling pathway.^26^ Breast cancer cell-derived exosomes induce CAFs activation through miR-130b-3p and its target SPIN90.^27^ The exosomal miR-1247-3p derived from liver cancer cells induces CAFs activation to facilitate lung metastasis of liver cancer.^28^ There are also limited studies that demonstrate that growth factors released from cancer cells may activate CAFs. In high circ_0020256 expression cholangiocarcinoma, cancer cell-secreted transforming growth factor β1 (TGF)-β1 promotes CAFs activation via Smad2/3 phosphorylation.^29^ TRAF6 in melanoma cells regulates the secretion of FGF19 through the NF-κB pathway, which activates CAFs to facilitate the malignant phenotype.^18^

IGF2 is reported to play an important role in tumor progression. IGF2 stimulates growth and metastasis of hepatocellular carcinoma through IGF1R and PTK2.^30^ IGF2 mediates the inhibition of exosomal miR-543 on the proliferation of ovarian cancer cells.^31^ Long non-coding RNA (LncRNA) MCF2L-AS1 contributes to cisplatin resistance of ovarian cancer via regulating the IGF2BP1/IGF2/MEK pathway.^32^ Furthermore, in a clinical study, Rasti et al. revealed that IGF2 was highly expressed in the urine of bladder cancer patients, suggesting IGF2 could be a potential biomarker for prognosis.^33^ Despite the intensive studies on the role of IGF2 in cancer, whether IGF2 is responsible for the activation of CAFs is not fully elucidated. Xu et al. reported that when inhibitor of differentiation (Id1) was overexpressed in esophageal cancer cells, esophageal cancer cells secreted IGF2 to activate fibroblasts, and CAFs facilitated VEGFR1-positive bone marrow cells to form pre-metastatic niches.^20^ It is worth to note that this study only indicated that the manipulated tumor cells may secrete IGF2 to activate CAFs; however, whether IGF2 secreted by primary cancer cells can activate fibroblast has not been evaluated. In our study, we found that lung cancer cells reprogramed NFs into CAFs through IGF2 secretion, demonstrated by applying recombinant IGF2, IGF2 neutralizing antibody, and IGF2 knockdown.

Autophagy is an intracellular self-degradative process, which helps to maintain cellular homeostasis. Autophagy status is critical for CAFs functions and influences their effects on tumor cells such as EMT, senescence, stemness, drug resistance, and metastasis.^15^ Increased autophagy of CAFs stimulates bladder cancer cell proliferation, invasion, and aerobic glycolysis.^34^ Our previous study demonstrated that high autophagy level of CAFs is pivotal for the autophagic secretion of HMGB1, which promoted the metastasis of lung cancer cells.^10^ On the contrary, inhibition of CAFs’ autophagy impairs cytokine secretion from fibroblasts and fibroblasts’ effect on collagen architecture, extracellular matrix stiffening, and tumorigenesis.^35^ Zhang et al. reported that blocking autophagy enhanced the efficacy of anticancer drugs on pancreatic cancer cells both *in vitro* and *in vivo.*^36^

Since CAFs possess higher autophagy level than NFs^10^ and autophagy status is essential for CAFs function, this drove us to explore whether autophagy induction is required for CAFs activation. We found that lung cancer cells induced autophagy of NFs, and the employment of autophagy agonist RAPA and antagonist 3-MA further demonstrated that autophagy induction mediated CAFs activation. Interestingly, Huang et al. reported that they treated NIH3T3 fibroblast cells with H_2_O_2_ to cause oxidative stress; when they further added TGF-β1 to these cells, they found that TGF-β1 could convert H_2_O_2_-treated NIH3T3 cells into CAF-like cells, and this was partially mediated by autophagy induction.^37^ In addition, a recent *in vivo* study showed that when genetically modified pancreatic ductal adenocarcinoma cells were transplanted into conditional whole-body ATG5-deficient mice, the transformation of quiescent pancreatic stellate cells to CAFs was attenuated in autophagy-deficient hosts, indicating the critical role of autophagy in CAFs activation.^38^ Although these studies did not investigate the role of autophagy in cancer cell-induced CAFs activation, their results well supported our findings.

CAFs have been documented to promote cancer cell growth and invasion. To verify whether iCAFs function similarly as CAFs, the effects of iCAFs on lung cancer cell proliferation and motility *in vitro* and tumor growth *in vivo* were determined and compared with CAFs. We found that iCAFs exerted similar pro-tumor function as CAFs. CAFs facilitate tumor development via secreting growth factors and cytokines. CXCL12 is a stromal cell-derived chemokine, playing diverse roles in cellular functions including tumor growth and metastasis. Several studies have shown that CAFs promote tumor progression via releasing CXCL12. CAFs-secreted CXCL12 induced EMT and cisplatin resistance in epithelial ovarian cancer though CXCR4/Wnt/β-catenin pathway.^22^ CD248-expressing CAFs-derived CXCL12 mediated M2-polarized macrophages and promoted lung cancer progression.^23^ Our results demonstrated that iCAFs facilitated lung cancer cell growth and invasion via CXCL12 secretion. AKT/NF-κB pathway is involved in tumorigenesis and tumor development and is reported to be a downstream signaling pathway of CXCL2. Wang et al. showed that CXCL12 enhances the resistance of chronic myelogenous leukemia cells to Adriamycin by increasing the expression of CXCR4 and activating the downstream AKT/NF-κB pathway.^39^ Xiao et al. reported that Flavokawain A inhibits the vasculogenic mimicry of hepatocellular carcinoma by suppressing CXCL12-mediated AKT/NF-κB signaling pathway.^40^ Consistent with these findings, our study revealed that iCAFs enhance lung cancer cell migration and invasion via CXCL12-mediated AKT/NF-κB pathway.

In summary, we demonstrated that lung cancer cells activated CAFs via IGF2-mediated autophagy induction and, in return, iCAFs stimulated lung cancer cell migration and invasion via CXCL12-mediated AKT/NF-κB pathway, indicating a positive feedback regulation between lung cancer cells and CAFs. Our finding provides a new insight for lung cancer therapy by targeting IGF2-mediated autophagy induction.

### Limitations of the study

It is worth to note that, when comparing the promoting effects on lung cancer cell functions including cell proliferation, colony formation, migration, and invasion, CAFs were slightly more effective than iCAFs. This finding suggested that our *in vitro* CAFs activation model did not fully mimic the *in vivo* situation, implying that there are other genes and mechanisms that are involved in CAFs activation.

In addition, the IGF2 validation experiments showed that the enhancing effects of iCAFs and CAFs on cell migration and invasion seemed stronger than IGF2, indicating that although IGF2 mediated the effect of iCAFs and CAFs on lung cancer cells invasion, some other factors might be also responsible for this. Thus, an in-depth understanding of the interaction between cancer cells and CAFs is urgently needed.

## Resource availability

### Lead contact

Further information and requests for resources and reagents should be sent directly to the lead contact, Ke Xu (ke_xu@hotmail.com).

### Materials availability

This study did not generate any new unique reagents, and all materials in this study are commercially available.

### Data and code availability

• Data: all data reported in this paper will be shared by the lead contact upon request.
• Code: this paper does not report original code.
• All other requests: any additional information required to reanalyze the data reported will be shared by the lead contact upon request.

## Acknowledgments

This work was supported by grants from the 10.13039/501100001809National Natural Science Foundation of China (81372519), the Key Project of Natural Science Foundation of Tianjin (22JCZDJC450), the Project of Tianjin Municipal Health Commission (TJWJ2021MS006), the Tianjin Key Medical Discipline (Specialty) Construction Project (TJYXZDXK-061B), and the Project of Tianjin Municipal Education Commission (2020KJ150, 2021KJ212).

## Author contributions

K.X. conceived, planned, and supervised the project; K.X. and L.C. designed experiments; L.C., B.L., S.Z., Q.Z., Y.Q., Y.R., H.W., and J.Z. performed experiments and analyzed results; L.C., X.W., and K.X. wrote the manuscript. All authors read and approved the final manuscript.

## Declaration of interests

The authors declare no competing interests.

## STAR★Methods

### Key resources table

**Table:** 

REAGENT or RESOURCE	SOURCE	IDENTIFIER
**Antibodies**		

AKT	Cell Signaling Technology	Cat#4685; RRID:AB_2225340
p-AKT	Cell Signaling Technology	Cat #4060; RRID:AB_2315049
E-cadherin	Cell Signaling Technology	Cat#3195; RRID:AB_2291471
MMP2	Cell Signaling Technology	Cat#40994; RRID:AB_2799191
MMP9	Cell Signaling Technology	Cat#15749; RRID:AB_2923397
p65	Cell Signaling Technology	Cat#8242; RRID:AB_10859369
ATG5	Cell Signaling Technology	Cat#9980S; RRID:AB_10829153
LC3	Cell Signaling Technology	Cat#12741; RRID:AB_2617131
Vimentin	Cell Signaling Technology	Cat#5741; RRID:AB_10695459
α-SMA	Cell Signaling Technology	Cat#19245; RRID:AB_2734735
FAP	Cell Signaling Technology	Cat#66562; RRID:AB_2904193
N-cadherin	Cell Signaling Technology	Cat#13116; RRID:AB_2687616
p-p65	Wanleibio	Cat#WL02169; RRID:AB_331284
CXCR4	Immunoway	Cat#YN5620; RRID:AB_2800286
HRP goat anti-rabbit IgG	ZSGB-BIO	Cat#ZB-2301; RRID:AB_2747412
HRP goat anti-mouse IgG	ZSGB-BIO	Cat#ZB2305; RRID:AB_2747415
IGF2 neutralizing antibody	R&D Systems	Cat#AF-292-NA; RRID:AB_354449
CXCL12 neutralizing antibody	R&D Systems	Cat#MAB310; RRID:AB_2276927

**Chemicals, peptides, and recombinant proteins**		

3-methyladenine	Sigma-Aldrich	Cat#5142-23-4
Acridine orange	Sigma-Aldrich	Cat#65-61-2
Rapamycin	Sigma-Aldrich	Cat#53123-88-9
Perifosine	Topscience	Cat#157716-52-4
JSH-23	Topscience	Cat#749886-87-1
Matrigel	Corning	Cat#356234
Trans-well	Corning	Cat#3422
Recombinant human IGF2	Sino Biological	Cat#13032-HNAY
Recombinant human CXCL12	Sino Biological	Cat#10118-H01H
ELISA kits for human IGF2	R&D system	Cat#DG200
ELISA kits for human CXCL12	Jiangsu Meimian Industrial	Cat#MM-0193H1

### Experimental model and study participant details

#### Ethics approval and consent to participate

This study has received the permission from the Ethical Review Committee of Tianjin Medical University General Hospital. The consents were obtained from the patients.

#### Cell lines

Lung cancer cell lines A549, H1299, H661 were purchased from the American Type Culture Collection (Manassas, VA), and they were mycoplasma free.

#### Mice

Five to six weeks old BALB/C-nude mice were purchased from the Chinese Academy of Medical Science (Beijing, China). Animal experiments were in accordance with protocols approved by the Animal Care and Experiment Committee of Tianjin Medical University General Hospital.

### Method details

#### Cell culture

A549 cells were cultured in DMEM (GIBCO, Grand Island, NY), H1299 and H661 cells were maintained in RPMI1640 with 10% fetal bovine serum and 1% penicillin-streptomycin (GIBCO).

Normal fibroblasts (NFs) and cancer-associated fibroblasts (CAFs) were isolated from adjacent non-tumor tissues and tumor tissues of NSCLC patients. NFs and CAFs were grown in DMEM/F12 with 10% fetal bovine serum and 1% penicillin-streptomycin. For the collection of conditioned medium (CM), NFs or CAFs were cultured in DMEM/F12 medium supplemented with 10% FBS, and the CM was collected after 48 h.

#### Cell proliferation assay

Cell viability was detected by using the Cell Counting Kit-8 (CCK8, Dojindo, Kumamoto, Japan). NFs cells were plated at a density of 3 × 10^3^ cells/well in a 96-well plate, lung cancer cells were plated at a density of 4-6 × 10^3^ cells/well in a 96-well plate, and then were reacted for 48 h. The absorbance at A450 was measured.

#### Colony formation assay

Lung cancer cells (1×10^3^/well) were seeded in 12-well plates with normal medium or CM. Medium was changed every two days for 10-14 days. Then cells were fixed with 4% paraformaldehyde (PFA) and stained with 0.1% crystal violet solution for 15 min. The images were collected by a scanner.

#### Cell invasion assay

Cell invasion ability was examined by trans-well assay. Briefly, the matrigel was coated on the upper chamber of the trans-well for 1 h in an incubator. Cells (5x10^4^) were added to the upper chamber in normal medium, and CM was added to the lower chamber. After 48 h cells were fixed in 4% PFA. The invaded cells in the lower chamber were stained with 0.1% crystal violet solution for 15 min, then were counted from 5 random fields under a microscope.

#### Cell migration assay

Cell migration was assessed by the wound healing assay. Cells were grown in 6-well plates until reached 100% confluence. A sterile pipette tip was used to scratch the cell monolayer, and then cells were cultured for indicated time points. The images were collected using a microscope at a magnification of 40x, and the closure rate was calculated. NFs migration was assessed by trans-well assay without matrigel coating.

#### Enzyme-linked immunosorbent assays (ELISA)

The ELISA kits for human IGF2 and CXCL12 were used to detect IGF2 and CXCL12 concentration. Cell culture medium was collected and cytokine levels were detected according to the kit’s instructions.

#### Immunofluorescence staining

Cells were fixed in 4% PFA for 20 min, and treated with 0.25% Triton X-100 for 15 min. After blocking with 5% BSA for 1h, cells were incubated with primary antibody overnight at 4°C, and then were incubated with fluorescein secondary antibody for 1h. Nucleus was stained with DAPI, and protein expression was observed under a fluorescence microscope.

#### Detection of acidic vesicular organelles (AVOs)

The formation of AVOs was detected by AO staining. Briefly, NFs were seeded in a 12-well plate at a density of 4×10^4^ cells/well and treated with different CM. Cells were stained with 0.5 μg/mL of AO for 15 min, then observed under a fluorescence microscope.

#### Quantitative PCR (qPCR)

Briefly, RNA was extracted from cells by using Trizol. Reverse transcription was performed using a Takara kit. Gene expression was analyzed using the Power SYBR Green Master Mix on an ABI Prism 7900 Sequence Detector System. GAPDH was used as an internal control. The primers are listed in the table below.

**Table:** PCR primer sequences

Primers	Sequences (5′-3′)		Length of amplicons (bp)
GAPDH	forward	TGCACCACCAACTGCTTAGC	87
	reverse	GGCATGGACTGTGGTCATGAG	
MMP2	forward	GCGGCGGTCACAGCTACTT	71
	reverse	CACGCTCTTCAGACTTTGGTTCT	
IGF2	forward	CGGACAACTTCCCCAGATAC	191
	reverse	GTCTTGGGTGGGTAGAGCAA	
α-SMA	forward	CACTGCCGCATCCTCATC	161
	reverse	TGCTGTTGTAGGTGGTTTCAT	
FAP	forward	GAAAGAAAGGTGCCAATA	116
	reverse	GATCAGTGCGTCCATCA	
CXCL12	forward	GAAGCCCGTCAGCCTGAG	162
	reverse	TTCGGGTCAATGCACACTTG	
MMP9	forward	CGACCAGGACAAGCGTTATG	124
	reverse	ACAGGCAGAGTACTCCTTGC	
E-cadherin	forward	AAGTGCTCTTCCAG	79
	reverse	GCGGCATTGTAGGTGT	
Vimentin	reverse	TTCCTCCCTGAACCGA	121
	forward	AGTTTCGTTGATAACCTGTCC	

#### Western blotting

Cells were lysed using RIPA buffer containing protease inhibitor. Proteins were separated by 12% SDS-PAGE and transferred onto nitrocellulose membranes. The membranes were blocked in Tris-buffered saline (TBS) with 5% milk and 0.1% Tween for 1 h. After probed with primary antibodies at 4°C overnight, membranes were incubated with HRP-conjugated secondary antibodies. The membranes were visualized using ECL solution on a G:BOX iChemi XT system (Syngene, Cambridge, UK).

#### *In vivo* xenograft mouse model

The mice were distributed into four groups consisting of A549 cells alone, A549 cells + NFs, A549 cells + iCAFs (induced CAFs), A549 cells + CAFs. Cells were subcutaneously injected into the flank of nude mice. Tumor volumes and mice body weight were measured every week. The tumor volume was calculated as follow: Volume = *d*^*2*^ *× D/2*, where *D* is the longest diameter and *d* is the shortest diameter. When the experiment was terminated after 7 weeks, tumor tissues were collected for further examinations.

### Quantification and statistical analysis

The data was presented as mean ± standard deviation, and were obtained from at least three independent experiments. The comparison analysis was performed using a one-way analysis of variance (ANOVA) for three or more groups, or using Student's *t* test for two groups. *P < 0.05* was defined as statistically significance.
